# Diagnostic Value of Conjunctival Impression Cytology and Nelson Grading in Evaluating Dry Eye Disease in Ankylosing Spondylitis

**DOI:** 10.3390/medicina61122147

**Published:** 2025-12-01

**Authors:** Tulay Yildirim, Seyhan Dikci, Ayse Nur Akatli

**Affiliations:** 1 Department of Physical Medicine and Rehabilitation, Inonu University Turgut Özal Medical Center, 44280 Malatya, Turkey; 2 Department of Ophthalmology, Aydın University, 34295 Istanbul, Turkey; seyhandikci@gmail.com; 3 Department of Pathology, Inonu University Turgut Özal Medical Center, 44280 Malatya, Turkey; aysenurakatli@gmail.com

**Keywords:** ankylosing spondylitis, dry eye disease, impression cytology, Nelson classification system

## Abstract

*Background and Objectives*: Ankylosing spondylitis (AS) is a chronic inflammatory disorder frequently associated with acute anterior uveitis; however, it may also predispose individuals to dry eye disease. We aimed to evaluate dry eye in AS participants employing both conventional tests and conjunctival impression cytology (Nelson grading) to compare their sensitivity in detecting ocular surface changes. *Materials and Methods*: This prospective case–control study enrolled 24 patients with AS and 27 age- and sex-matched healthy controls. Dry eye was evaluated using the Schirmer I test (measuring tear production), fluorescein tear break-up time (BUT, assessing tear film stability), the Ocular Surface Disease Index (OSDI) questionnaire (evaluating symptoms), and conjunctival impression cytology (analyzing ocular surface changes). Intergroup differences were assessed using non-parametric statistical methods, and their correlations with clinical variables were examined. *Results*: Nelson conjunctival cytology scores were significantly higher in AS patients than control subjects (1.63 ± 0.92 vs. 0.74 ± 0.86; *p* = 0.001), indicating greater conjunctival goblet cell loss and squamous metaplasia. In contrast, Schirmer, BUT, and OSDI observations did not differ significantly between AS and control subjects (all *p* > 0.05). Within AS, the Nelson cytology grade did not correlate accompanied by tear test observations or disease activity indices, and symptom scores did not align accompanied by Schirmer or BUT values. *Conclusions*: AS patients demonstrated evidence of subclinical dry eye changes detectable by impression cytology even when standard tests were normal. Conjunctival cytology was more sensitive than Schirmer, BUT, or symptom assessment in identifying ocular surface involvement in AS. Integrating such ocular surface evaluation into AS management could allow earlier diagnosis of dry eye and prompt intervention to improve patient quality of life.

## 1. Introduction

AS is a chronic immune-mediated inflammatory disease predominantly affecting the axial skeleton, with potential extra-articular manifestations involving multiple organ systems. Ocular involvement represents one of the most frequent extra-articular manifestations of AS, with acute anterior uveitis developing in approximately 25–40% of patients during their disease course and recognized as the hallmark ocular complication. In addition to uveitis, various other ocular problems (e.g., ptosis, superficial keratitis, episcleritis, scleritis, corneal ulcers) have been reported in AS, reflecting the broad spectrum of its effects on eye health. Recently, dry eye disease has also been highlighted as part of the ocular manifestations of spondyloarthritis [1,2].

Emerging evidence suggests that patients with AS exhibit an elevated risk of developing dry eye disease (keratoconjunctivitis sicca). Chronic systemic inflammation in AS is thought to affect the lacrimal glands and ocular surface, potentially disrupting the tear film’s stability and leading to dry eye symptoms. Indeed, several studies have demonstrated that patients with AS exhibit significantly greater tear film instability, evidenced by shorter tear break-up times (TBUT), and potentially reduced tear secretion compared to healthy controls, indicating a higher prevalence of dry eye disease (DED) in this population. Dry eye disease (DED) is a multifactorial condition of the ocular surface characterized by insufficient tear production and/or rapid tear evaporation, leading to ocular discomfort and potential surface damage. In AS, ongoing inflammation may induce structural and functional changes in the lacrimal functional unit, exacerbating or precipitating dry eye. Dry eye in AS is clinically significant as it may impair patients’ quality of life due to chronic ocular discomfort and poses a risk of ocular surface damage if left undiagnosed.

Overall, however, the current literature on dry eye in AS remains limited and sometimes inconsistent. Although some studies have reported no significant differences in dry eye parameters between AS patients and healthy controls, larger-scale studies have consistently demonstrated significant impairments in tear secretion, tear film stability, and ocular surface integrity in AS patients [2,3,4]. Notably, a recent international multicenter registry study demonstrated that patients with axial spondyloarthritis (including AS) exhibited a significantly higher prevalence of dry eye symptoms, with approximately a ninefold increased odds of dry eye disease compared to healthy controls [5]. Similarly, a large population-based cohort study demonstrated that AS was associated with a nearly twofold increased risk of clinically diagnosed DED compared to non-AS individuals [6]. These inconsistent findings underscore the necessity of additional studies to elucidate the precise prevalence and clinical characteristics of dry eye disease in AS, as well as to determine the optimal diagnostic modalities for detecting ocular surface abnormalities in this patient population. We define dry eye disease (DED) according to the TFOS DEWS II criteria as a multifactorial ocular surface disorder characterized by symptoms, tear-film instability and/or hyperosmolarity, with ocular surface inflammation and damage, whereas “ocular inflammation” denotes clinically apparent entities (e.g., anterior uveitis, scleritis, episcleritis, keratitis) detected at slit-lamp and typically requiring anti-inflammatory therapy.

Given these considerations, we conducted this study to assess dry eye disease in patients with AS, using both subjective and objective diagnostic measures, as well as to evaluate the diagnostic utility of conjunctival impression cytology (graded according to the Nelson classification system) in this clinical setting. By comparing AS patients with healthy controls, we aimed to determine whether conjunctival cytology can identify subclinical ocular surface inflammation in AS that may not be detected by conventional dry eye tests, including Schirmer test, BUT, and the OSDI.

## 2. Methods

### 2.1. Study Design and Participants

This prospective, cross-sectional case–control study compared patients with ankylosing spondylitis (AS) and age- and sex-matched healthy controls diagnosed according to the modified New York criteria. Patient enrollment and data collection were planned in advance, all assessments were performed at a single time point, and both groups were evaluated comparatively. The modified New York criteria require radiographic sacroiliitis (grade ≥ 2 bilaterally or grade 3–4 unilaterally) plus at least one clinical criterion, including low back pain and stiffness for more than 3 months that improves with exercise, limitation of lumbar spine motion, or reduced chest expansion. Disease-specific parameters, including disease duration, serum C-reactive protein (CRP) levels, erythrocyte sedimentation rate (ESR), and Bath Ankylosing Spondylitis Disease Activity Index (BASDAI) scores, were documented for AS patients.

### 2.2. Exclusion Criteria

The exclusion criteria were established to ensure that the observed ocular findings were specifically attributable to AS. Patients presenting with acute anterior uveitis flare or other clinically significant ocular pathologies (unrelated to dry eye disease) at the time of screening were excluded. We excluded participants with active anterior uveitis or other overt ocular inflammatory diseases at screening (keratitis, episcleritis, or scleritis), prior ocular surface surgery, systemic diseases associated with primary DED (e.g., Sjögren’s), contact lens wear, or current pregnancy. This exclusion was specifically applied to differentiate subclinical ocular surface alterations attributable to AS from clinically apparent ocular inflammatory events. Therefore, none of the included eyes had active inflammation at the time of evaluation. All participants provided written informed consent prior to enrollment, and the study protocol was approved by the local institutional review board (IRB) in compliance with the principles of the Declaration of Helsinki.

### 2.3. Dry Eye Assessments

All subjects underwent a standardized dry eye evaluation by the same ophthalmologist in the following order:

- The Schirmer I test is performed without anesthesia to assess baseline tear secretion. A sterile Schirmer strip (5 × 35 mm) was placed at the lateral lower eyelid margin of each eye. After 5 min, the length of strip wetting was measured (mm). The mean value of both eyes was calculated. Conventionally, ≤5 mm of wetting in 5 min is indicative of severe dry eye, 6–10 mm is borderline, and >10 mm is normal.
- Tear Film Break-Up Time (BUT): Assessed tear film stability. A drop of 1% fluorescein dye was instilled into the inferior fornix of each eye. After a few blinks, the patient exhibited a fixed gaze with cessation of blinking, and corneal examination was performed under cobalt blue light. The interval between the last blink and the appearance of the first corneal dry spot was measured using a stopwatch. Three measurements were taken per eye and averaged; a BUT < 10 s was considered abnormal (indicative of tear film instability).
- Ocular Surface Disease Index (OSDI): Participants completed the OSDI questionnaire, which quantifies the frequency of dry eye symptoms and their impact on daily activities (score range 0–100; higher scores indicate more severe symptoms). We recorded each subject’s overall OSDI score.
- Conjunctival Impression Cytology: Conjunctival impression cytology specimens were stained with periodic acid-Schiff (PAS) and hematoxylin-eosin (H&E) and graded by a masked ophthalmic pathologist using the Nelson system: Grade 0 (normal goblet cells/morphology), Grade 1 (mild squamous metaplasia with some goblet cell loss), Grade 2 (moderate metaplasia with marked goblet cell loss), Grade 3 (severe metaplasia with few/absent goblet cells). Conjunctival impression cytology detects goblet-cell loss and squamous metaplasia—microscopic hallmarks of ocular-surface stress—providing complementary information to tear quantity and stability tests. When interpreted alongside Schirmer, TBUT, and the clinical exam, IC assists in recognizing subclinical DED-spectrum changes in AS after exclusion of overt inflammatory eye disease.

### 2.4. Statistical Analysis

Statistical analyses were conducted using IBM SPSS Statistics, Version 22.0 (IBM Corp, Armonk, NY, USA). For group comparisons (AS vs. control), continuous variables were expressed as mean ± standard deviation (median) and analyzed using the Mann–Whitney U test due to non-normal distributions, whereas categorical variables (e.g., sex) were compared using chi-square tests. Within the AS group, correlations between ocular parameters (Schirmer’s test, BUT, OSDI, and Nelson grade) and clinical parameters (disease duration, CRP, ESR, and BASDAI) were evaluated using Spearman’s rank correlation coefficient (ρ). A *p*-value < 0.05 was considered statistically significant for all analyses.

## 3. Results

### 3.1. Participant Characteristics

The study cohort comprised 24 patients with AS (16 male, 8 female) and 27 age-matched healthy controls (12 male, 15 female). The mean age was 41.0 ± 9.4 years in the AS group and 42.4 ± 13.8 years in the control group. No significant differences were observed between the groups regarding age or sex distribution (*p* > 0.05), indicating adequate matching of baseline demographic characteristics.

Among the AS patients, the mean disease duration since diagnosis was 4.96 ± 3.53 years (median: 3.5 years). The average CRP level was 0.86 ± 0.65 mg/dL (median 0.5), and the mean ESR was 19.7 ± 15.4 mm/h (median 14). These values reflect a range of inflammatory activity at the time of ocular evaluation. The mean BASDAI score was 3.75 ± 1.61 (median 3.6), indicating moderate disease activity on average. Some AS patients were on conventional therapies, including nonsteroidal anti-inflammatory drugs (NSAIDs) or disease-modifying antirheumatic drugs (DMARDs), with no active uveitis flare observed during the study period.

### 3.2. Dry Eye Test Outcomes (AS vs. Control)

Table 1 summarizes the key ocular surface parameters in the AS and control groups. The most significant difference was noted in the conjunctival impression cytology findings. AS patients exhibited significantly greater conjunctival epithelial alterations indicative of dry eye disease compared to controls. The mean Nelson grading score in the AS group was 1.63 ± 0.92, compared to 0.74 ± 0.86 in the control group (*p* = 0.001). In clinical practice, a significant proportion of AS patients exhibited moderate conjunctival squamous metaplasia (Nelson grade 2), whereas the majority of controls demonstrated normal conjunctival epithelium or only mild pathological changes. This finding suggests that subclinical ocular surface inflammation—characterized by goblet cell loss and epithelial morphological changes—is present in AS patients, even in the absence of overt dry eye signs or symptoms.

In contrast, conventional clinical dry eye tests failed to demonstrate statistically significant differences between the two cohorts. Schirmer I test: The mean Schirmer test value was 11.7 ± 9.4 mm in AS patients and 12.6 ± 7.6 mm in the control group, with no statistically significant difference between the groups (*p* = 0.416). These findings indicate comparable baseline tear production in both cohorts. TBUT: analysis revealed a mean of 17.8 ± 9.6 s in AS patients compared to 19.1 ± 9.4 s in the control group (*p* = 0.455), demonstrating no statistically significant difference in tear film stability between the two cohorts. There was no significant difference in OSDI symptom scores between the AS patients and controls, with mean scores of 19.3 ± 17.4 and 21.1 ± 14.0, respectively (*p* = 0.497). Within each group, symptom scores exhibited a wide range (from asymptomatic individuals to those with moderate symptoms); however, the AS group overall did not demonstrate significantly worse dry eye symptoms compared to the control group. Although the mean OSDI score in the AS group was slightly lower (indicating better outcomes) compared to the control group, this difference did not reach statistical significance.

In summary, apart from the conjunctival cytology findings, the AS patients demonstrated no significant dry eye abnormalities on conventional diagnostic tests or symptom-based questionnaires relative to the healthy controls. This suggests that many AS patients in our cohort had early-stage or mild dry eye disease that was not detectable by Schirmer’s test, BUT, or OSDI, despite the presence of microscopic ocular surface alterations.

### 3.3. Correlation Analyses in AS

As demonstrated in Table 2, within the AS group, Schirmer test values exhibited a moderate positive correlation with BUT (Spearman’s ρ = 0.579, *p* = 0.003), suggesting that AS patients with greater tear secretion also displayed increased tear film stability. Schirmer’s test also showed a weak positive correlation with ESR (ρ = 0.407, *p* = 0.048), whereas no significant correlations were observed between Schirmer’s test and CRP or BASDAI. Importantly, the conjunctival cytology grade (Nelson score) did not show a significant correlation with any other dry eye parameters (Schirmer test, BUT, or OSDI) or with systemic inflammatory markers. In other words, severe conjunctival changes (high Nelson grade) did not correlate with low tear production, tear film instability, or symptom severity—some AS patients with normal tear test results exhibited marked conjunctival changes, while others with abnormal tear parameters had a low Nelson grade.) Similarly, OSDI symptom scores showed no significant correlation with either Schirmer test results or TBUT (both *p* > 0.05), highlighting the frequent discrepancy between subjective symptom reports and objective tear film parameters. No meaningful correlations were observed between any ocular parameter and the BASDAI disease activity score or CRP level in the AS group.

## 4. Discussion

In this study, we conducted a systematic evaluation of dry eye disease manifestations in patients with AS, employing both standard dry eye diagnostic tests and conjunctival impression cytology. Our findings demonstrate that the effect of AS on the ocular surface may be subclinical and could evade detection by conventional diagnostic modalities in early-stage disease. Notably, we observed significant conjunctival cellular alterations in AS patients despite largely normal average Schirmer test scores, BUT, and OSDI results. The lack of statistically significant differences in Schirmer test, BUT, and OSDI scores between the AS group and the control group—despite evident cytological abnormalities—highlights the greater sensitivity of impression cytology in detecting early subclinical ocular surface alterations in AS patients. This suggests that AS-related ocular surface involvement might begin at a microscopic, subclinical level, and traditional tests might only become abnormal at more advanced stages of damage.

The significantly higher Nelson grading scores in AS patients suggest more severe conjunctival squamous metaplasia and goblet cell depletion, reflecting chronic ocular surface inflammation in this population. This result aligns accompanied by previous reports. Oltulu et al. (2019) also found significantly elevated conjunctival impression cytology scores in AS participants compared to control subjects, mirroring our observations [7]. Our study corroborates those observations in a smaller cohort, emphasizing that even in the absence of obvious clinical dry eye, AS can affect the conjunctival epithelium at a cellular level.

Conjunctival impression cytology is uniquely valuable in uncovering this “silent” inflammation—whereas Schirmer and BUT provide information on tear quantity and stability, cytology directly visualizes cellular alterations on the ocular surface. In a systemic inflammatory disease like AS, immune-mediated changes (e.g., lymphocytic infiltration, inflammatory cytokines) in the conjunctiva and lacrimal functional unit can occur before tear production is significantly reduced [8]. Indeed, in our cohort, many patients with AS had normal Schirmer test and BUT results but exhibited Nelson grade 2 changes on conjunctival impression cytology. This finding suggests that impression cytology can detect early ocular involvement in AS that might otherwise go unnoticed. Early recognition of these microscopic ocular surface alterations is clinically important even in the absence of overt symptoms, as preventive interventions—such as lubrication, environmental adjustments, and topical anti-inflammatory therapy—may halt or slow disease progression. Although no curative therapy currently exists for dry eye disease, early detection helps maintain ocular comfort and surface integrity, and may become even more relevant as biomarker-based and disease-modifying treatments evolve in the future. In other words, this subclinical “silent” stage provides a crucial window for early intervention. Patients accompanied by high Nelson grades—even if asymptomatic or only mildly symptomatic—should be flagged for closer monitoring and prompt treatment. We propose that if an AS patient demonstrates an abnormal cytology result (e.g., Nelson grade 2–3) despite normal traditional tests, they should be considered as having subclinical dry eye and managed proactively. Initiating prophylactic treatments such as lubricating eye drops or topical anti-inflammatory drops might help prevent progression of ocular surface damage.

On the other hand, the absence of group-level differences in Schirmer and BUT in our study deserves discussion. At first glance, this may seem inconsistent accompanied by some prior literature that reported tear production or tear quality deficits in AS. However, several factors likely contribute. Our AS participants were relatively young (mostly in their 30 s and 40 s) accompanied by a moderate disease duration (~5 years) and moderate disease activity. It is possible that significant lacrimal gland impairment or meibomian gland dysfunction had not yet developed in many of these participants. In contrast, studies involving participants accompanied by longer-standing or more severe AS have found clearer evidence of dry eye. Roda et al. (2020) and Oltulu et al. (2019) both documented substantially lower Schirmer values and shorter BUT in AS participants compared to control subjects, especially in those accompanied by more active or longstanding disease (Table 3) [3,7].

Unlike those studies, we did not observe a significant reduction in tear production or stability in our AS cohort. Instead, our Schirmer and BUT values were similar to control subjects (*p* > 0.05), suggesting that not every AS patient will manifest overt dry eye signs, particularly if their systemic disease is moderately controlled or of shorter duration. Some AS participants might have what could be termed “occult” or latent dry eye—early ocular surface changes that are compensated by reflex tearing or not yet severe enough to reduce tear production. This could explain why our AS group’s average tear metrics were similar to control subjects even though cytology indicated underlying changes. It highlights the importance of considering subclinical disease: a lack of abnormal observations on Schirmer/BUT does not guarantee that the ocular surface is completely healthy in AS.

The lack of difference in OSDI symptom scores between our AS participants and control subjects also suggests that many AS participants did not experience pronounced dry eye symptoms at the time of evaluation. This could be attributable to early-stage involvement as well—participants might simply not notice mild dryness, or they may attributable minor symptoms to other causes. In a larger survey, axial spondyloarthritis participants did report significantly more dry eye symptoms than control subjects (roughly 65% vs. 16%) [5], so symptom prevalence can vary by population. It is also known that dry eye symptoms are influenced by individual factors and can fluctuate. Environmental factors (e.g., climate, screen time) and comorbid conditions (allergies, etc.) can modulate symptom reporting. Moreover, AS participants might be more focused on their musculoskeletal discomfort and therefore less likely to mention mild eye irritation unless specifically questioned. Another intriguing possibility is that some AS participants may have neuropathic alterations or reduced corneal sensation (due to chronic inflammation) that make them less sensitive to dryness, or conversely, some may have heightened pain sensitivity. This concept is supported by recent in vivo confocal microscopy observations that AS participants have significantly reduced corneal nerve fibre densities compared to control subjects, suggesting subclinical neurotrophic changes in AS-related dry eye [9]. Our finding that objective signs did not align well accompanied by subjective symptoms is in line accompanied by the known discordance in dry eye: participants can have significant clinical observations accompanied by few symptoms, or severe symptoms accompanied by minimal clinical observations. Therefore, clinical vigilance is needed even in the absence of symptoms. For AS participants, it would be prudent to perform periodic eye examinations and not rely solely on patient complaints to trigger an evaluation [2].

The correlation analyses provided additional insights. The moderate correlation between Schirmer and BUT indicates that in our AS participants, tear production and tear film stability tended to improve or decline together—a logical interplay where adequate tear volume generally helps maintain tear film integrity. This relationship is well documented in dry eye pathophysiology; a reduction in tear secretion often leads to increased osmolarity and inflammatory changes that also degrade tear quality, creating a self-perpetuating cycle. An unexpected finding was the positive correlation between Schirmer and ESR. One might theorize that systemic inflammation could decrease tear secretion (as inflammatory cytokines can impair lacrimal gland function), however our data hinted at the opposite for ESR. We speculated that active inflammation might induce reflex tearing due to ocular surface irritation, temporarily elevating Schirmer observations. Another explanation could be sample-related—perhaps participants accompanied by longer disease (and thus higher cumulative inflammation and ESR) had developed compensatory mechanisms or minor gland fibrosis prompting reflex tearing. However, since CRP did not indicate a similar correlation accompanied by Schirmer, this observation should be interpreted cautiously. It might represent a spurious association or be due to the limited sample size. Further research is needed to clarify how systemic inflammatory markers relate to ocular parameters in AS.

Crucially, we observed no correlation between Nelson grades and any other dry eye tests. This suggests that conjunctival cellular changes progress somewhat independently of tear production and quality changes. In practical terms, an AS patient could have significant conjunctival metaplasia even if their Schirmer and BUT are normal, implying that impression cytology examines a different facet of ocular surface health than the tear-based tests. This is supported by observations in other conditions: for example, in some participants accompanied by early primary Sjögren’s syndrome, Schirmer observations can still be normal while conjunctival impression cytology already indicates goblet cell loss and squamous metaplasia. Thus, Nelson grading provides complementary information and can detect early ocular surface damage that traditional tests might miss.

From a clinical standpoint, our findings highlight the necessity of a comprehensive, multi-dimensional approach to ocular assessment in AS. If we had relied solely on patient symptoms or basic tear tests, we might conclude that most of our AS participants had no dry eye issues. However, conjunctival cytology revealed that many of them did have underlying pathology. Therefore, ophthalmologists and rheumatologists should be aware that AS participants may harbour “hidden” dry eye and ocular surface inflammation. We recommend that participants accompanied by AS undergo periodic ophthalmologic examinations even if they do not voice eye complaints [2]. Incorporating tests like impression cytology (or newer non-invasive imaging techniques, if available) could help identify those who would benefit from early interventions. In patients with abnormal impression cytology, clinicians may consider early, individualized ocular-surface management under ophthalmologic guidance; this approach warrants prospective confirmation and is not a blanket recommendation. Ensuring ocular surface integrity is part of improving the overall quality of life for AS participants, who are already burdened by chronic pain and stiffness. In essence, recognizing that ocular involvement in AS is not limited to uveitis however also includes surface disease broadens our scope of patient care. A multidisciplinary collaboration between rheumatology and ophthalmology is advisable for comprehensive management of AS, wherein rheumatologists remain vigilant for ocular symptoms and refer participants for eye evaluations even in the absence of acute uveitis.

Our findings raise hypotheses regarding systemic anti-inflammatory therapy and ocular-surface outcomes in AS. Some AS participants in our cohort were on anti-inflammatory medications (NSAIDs or DMARDs), which might have mitigated their dry eye signs. There is some evidence that biologic therapies, such as tumour necrosis factor (TNF) inhibitors, can positively influence ocular surface conditions in inflammatory diseases. Recent studies have indicated that AS participants treated accompanied by TNF inhibitors experience improvements in tear production and conjunctival cytology observations over time [10]. In one long-term study, AS participants on TNF-blockers had significant increases in Schirmer test values and a reversal of abnormal impression cytology to normal after 12 months of therapy, paralleling the improvement in systemic disease activity [10]. This suggests that effective control of systemic inflammation can also benefit ocular surface health—essentially, treating the underlying disease may treat the dry eye as well. (Conversely, rare cases of paradoxical dry eye or ocular surface side effects have been reported accompanied by certain biologics, though these are uncommon.) In our study we did not stratify participants by treatment, however the potential influence of medications is worth investigating in future research.) Ideally, larger longitudinal studies could determine whether aggressive control of AS inflammation (via biologics or other means) leads to better long-term ocular outcomes.

In summary, conjunctival impression cytology proved to be a valuable diagnostic tool in uncovering subclinical ocular surface changes in AS patients. To our knowledge, our study is one of the first from Turkey to employ this technique in AS, providing novel evidence that significant conjunctival metaplasia and goblet cell loss can be present even when standard dry eye tests (Schirmer, BUT, OSDI) are unremarkable. These observations suggest that routine eye assessments for AS should encompass not only symptom inquiry and basic tear tests, but also methods capable of detecting early microscopic changes. Thus, clinicians may identify at-risk participants sooner and initiate interventions to preserve ocular surface health. Given the potential for dry eye to impair vision and quality of life, proactive ocular monitoring and management should be integral to the care of individuals accompanied by ankylosing spondylitis.

### Limitations

This study has several limitations. The sample size was relatively small and drawn from a single centre, which may limit the generalisability of the observations. Additionally, the cross-sectional design provides only a snapshot of ocular condition accompanied without longitudinal follow-up, so we could not assess the progression of dry eye changes or the effects of treatment over time. We also did not stratify participants by medication utilize or disease severity, which could influence ocular surface parameters and tear metrics. This study had moderate average BASDAI and variable treatment regimens, which may have influenced ocular parameters. This limitation has been acknowledged and discussed; future longitudinal, treatment-stratified studies are recommended.

However, our study also has important methodological strengths that bolster the validity of the observations. It was prospectively designed accompanied by an age- and sex-matched control group, and all participants underwent the same standardized evaluation by a single experienced ophthalmologist, minimizing inter-observer variability. Furthermore, we employed strict inclusion and exclusion criteria (for example, excluding other causes of dry eye and any active ocular conditions unrelated to AS) to ensure that the ocular observations could be attributable specifically to AS. These measures increase confidence that the observed differences—particularly the conjunctival cytology observations—truly reflect AS-related ocular surface effects rather than confounding factors.

## 5. Conclusions

Patients with ankylosing spondylitis may exhibit subclinical ocular surface changes detectable by conjunctival impression cytology, even when conventional dry eye tests are normal. Conjunctival cytology appears more sensitive than Schirmer, BUT, or symptom assessment for identifying early ocular surface involvement. Integrating such microscopic evaluation into the routine management of ankylosing spondylitis could enable earlier recognition and treatment of dry eye disease, improving patients’ ocular health and quality of life. The inclusion of such early ocular surface screening may therefore serve as an important preventive strategy until curative options for dry eye disease become available.

### Key Messages

- Dry eye disease is an under-recognized ocular manifestation of AS, occurring even when participants have no eye symptoms.
- In this study, conventional dry eye tests (Schirmer I, tear break-up time, OSDI symp-tom scores) were largely normal in AS participants and not significantly different from healthy control subjects.
- Conjunctival impression cytology with Nelson grading revealed significant ocular surface changes, including goblet cell loss and squamous metaplasia, in AS patients, which were undetectable by conventional diagnostic methods.
- Conjunctival cytology appears more sensitive for early dry eye detection in AS; incorporating ocular surface screening into routine AS care may enable earlier intervention and better patient outcomes.

## Figures and Tables

**Table 1:** Demographic characteristics and dry eye parameters in patients with ankylosing spondylitis compared to healthy controls.

Parameter	Control Group (*n* = 27)	AS Patients (*n* = 24)	*p*-Value
Age (years)	42.37 ± 13.82 (40)	41.04 ± 9.42 (39.5)	0.68
Sex (M/F)	12/15 (44.4% male)	16/8 (66.7% male)	0.11
Schirmer I (mm/5 min)	12.56 ± 7.58 (12)	11.67 ± 9.37 (9)	0.42
Tear break-up time (TBUT, s)	19.11 ± 9.37 (17)	17.79 ± 9.61 (16)	0.46
OSDI score (0–100)	21.05 ± 14.01 (18.75)	19.26 ± 17.44 (11.93)	0.50
Nelson grade (0–3)	0.74 ± 0.86 (1)	1.63 ± 0.92 (2)	0.001 *

* *p* < 0.05 indicates statistically significant difference.

**Table 2:** Spearman’s correlation analysis of selected parameters in patients with AS.

Relationship (Parameter A–B)	Spearman’s ρ (Rho)	*p*-Value
Age–Disease duration (years)	0.474	0.019 *
Schirmer I–TBUT (s)	0.579	0.003 *
Schirmer I–ESR (mm/h)	0.407	0.048 *
Schirmer I–OSDI score	−0.106	0.622
Schirmer I–Nelson grade	0.028	0.897
Schirmer I–CRP (mg/dL)	–0.005	0.983
Schirmer I–Age	−0.060	0.780

* *p* < 0.05 indicates statistically significant correlation. Abbreviations: TBUT = tear break-up time; OSDI = Ocular Surface Disease Index; ESR = erythrocyte sedimentation rate; CRP = C-reactive protein.

**Table 3:** Comparison of dry eye observations in AS participants: present study vs. selected previous studies.

Study (Year)	Schirmer I (AS vs. Control)	TBUT (AS vs. Control)	OSDI Score (AS vs. Control)	Conjunctival Cytology (AS vs. Control)
Roda et al. (2020) [3]	AS < C (7.3 vs. 11.7 mm; *p* < 0.01) *	AS < C (7.3 vs. 14.0 s; *p* < 0.01) *	–	–
Oltulu et al. (2019) [7]	AS < C (12.2 vs. 20.3 mm; *p* < 0.001) *	AS < C (3.8 vs. 10.1 s; *p* < 0.001) *	AS > C (36.5 vs. 9.1; *p* < 0.001) *	AS > C (2.12 vs. 0.57; *p* < 0.001) *
This study (2025)	AS ≈ C (11.7 vs. 12.6 mm; *p* > 0.05)	AS ≈ C (17.8 vs. 19.1 s; *p* > 0.05)	AS ≈ C (19.3 vs. 21.1; *p* > 0.05)	AS > C (1.63 vs. 0.74; *p* = 0.001) *
Vitale et al. (2024) [1]	–	–	AS > C (higher OSDI; OR ~9.2)	–

AS = ankylosing spondylitis; C = control; TBUT = tear break-up time; OSDI = Ocular Surface Disease Index; OR = odds ratio; “–” indicates not measured. “AS < C” indicates the AS group’s value is significantly lower than that of control subjects, while “AS > C” indicates the value is significantly higher in AS. Statistically significant differences are indicated by an asterisk (*p* < 0.05). Reference [1] did not measure Schirmer I or TBUT; this multi-centre study reported significantly higher dry eye symptom scores in axial spondyloarthritis participants, corresponding to an approximately 9.2-fold increased odds of dry eye.

## Data Availability

The original contributions presented in this study are included in the article. Further inquiries can be directed to the corresponding author.
